# Radiation and cancer risk: a continuing challenge for epidemiologists

**DOI:** 10.1186/1476-069X-10-S1-S4

**Published:** 2011-04-05

**Authors:** Jonathan M Samet

**Affiliations:** 1 Department of Preventive Medicine, Keck School of Medicine of USC, USC Institute for Global Health, University of Southern California, 1441 Eastlake Ave. Room 4436, MC 9175, Los Angeles, CA 90089, USA

## Abstract

This paper provides a perspective on epidemiological research on radiation and cancer, a field that has evolved over its six decade history. The review covers the current framework for assessing radiation risk and persistent questions about the details of these risks: is there a threshold and more generally, what is the shape of the dose-response relationship? How do risks vary over time and with age? What factors modify the risk of radiation? The example of radon progeny and lung cancer is considered as a case study, illustrating the modeling of epidemiological data to derive quantitative models and the coherence of the epidemiological and biological evidence. Finally, the manuscript considers the need for ongoing research, even in the face of research over a 60-year span.

## Introduction

Ionizing radiation was discovered in 1895 by Wilhelm Conrad Roentgen, and its utility for diagnostic purposes was quickly recognized. By 1902, the first radiation-caused skin cancer was identified and the first radiation-caused leukemia case followed in 1911. Several clusters of radiation-caused cancer were described over the ensuing decades: radon and lung cancer in underground metal miners in eastern Europe, and osteogenic sarcoma in radium dial painters. In 1944, based on more formal epidemiological inquiry, an excess of leukemia was reported among radiologists in the United States [1]. By World War II, there was sufficient understanding of the risks of radiation to motivate a program of protection for workers at the Manhattan Project in the United States [2].

Radiation epidemiology was launched when a program of studies was initiated by the then Atomic Bomb Casualty Commission (eventually to become the Radiation Effects Research Foundation) to determine the consequences of radiation exposure from the nuclear blasts at Hiroshima and Nagasaki [3,4]. A large, prospective cohort was designed, the Lifespan Study of 120,000 survivors, which is still in progress. This cohort has proved to be a remarkably informative resource, providing a temporal profile of leukemia and cancer associated with the blast and a robust data set for making quantitative estimates of risk. Many cohort studies have followed with radiation exposures received through therapeutic intervention or occupation, or by accident. The resulting data base is extensive for several types of radiation including X and gamma radiation and radon. The data have been sufficient to support a radiation protection approach that is grounded in the epidemiological evidence (see below).

## Risk assessment, radiation, and cancer

**Figure 1:**
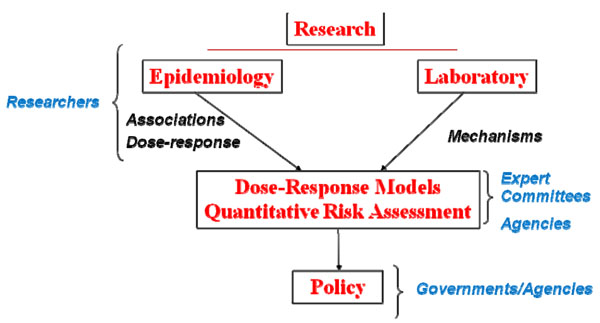
Evidence-based radiation protection

Radiation exposures are ubiquitous, coming from medical and industrial applications and from naturally occurring sources. Exposures are regulated through an evidence-based approach that is used to characterize risks, drawing primarily on the epidemiological evidence (Figure 1). Risk assessment is an applied methodology, used to characterize risks to populations as the basis for risk management [5]. A 1983 report of the United States National Research Council, *Risk Assessment in the Federal Government: Managing the Process* (widely referred to as the "red book") set out four elements of risk assessment: hazard identification (is there a problem?), dose-response assessment (how does risk vary with dose or exposure?), exposure assessment (what is the population's pattern of exposure?), and risk characterization (what is the magnitude of the problem and what are the key uncertainties in that understanding?) [6]. Many subsequent reports from the National Research Council and other groups have refined the elements of risk assessment, though the four components have proved to be invaluable in approaching risk questions. The framework is useful, not only for assembling evidence on risk, but for identifying evidence gaps, attendant uncertainties, and related research needs. Most recently, a National Research Council Committee gave emphasis to the need to make certain that questions were properly framed to assure that the findings of a risk assessment will prove valuable for risk management [5].

Risk assessment has become fundamental to strategies for limiting cancer risks associated with radiation exposures. There is a need to minimize risk at the population level and to assure that risks to individuals do not reach unacceptable levels, particularly for workplace exposures. Determination of the acceptability of risk requires an assessment of the magnitude of risk and a societal judgment as to acceptability of the estimated risk [7]. We are also learning that there is a spectrum of susceptibility to radiation that needs to be taken into account. Additionally, the millions of people receiving radiation for diagnostic and therapeutic purposes need to have an understanding of the attendant risks. Such medical exposures have now surpassed radon as the leading contributor to radiation exposure in the United States as use of diagnostic imaging has risen sharply [8].

For ionizing radiation, the principal uncertainty at present lies in the form of the dose-response relationship. There is no doubt as to the existence of a hazard and exposures are known with reasonable accuracy. However, at "low levels" uncertainty persists as to whether the dose-response relationship is linear and whether there is a dose threshold, below which there is no risk. Some have proposed that "low" exposures to radiation may be beneficial, a hypothesis referred to as "hormesis" [9]. This controversy is discussed subsequently in this manuscript. Over recent decades, various expert groups have adopted linear no-threshold dose-response models for radiation and cancer, based on review of epidemiological and biological evidence. This assumption is important because it assigns risk to any exposure and the burden of cancer attributable to radiation is consequently driven by the lower levels of exposure that contribute the bulk of the radiation dose to the population.

## Radon, epidemiology, and risk

Radon, a noble gas resulting from the decay of naturally occurring uranium-238, has been extensively studied using epidemiological methods, initially to characterize the risks faced by radon-exposed underground miners so as to promulgate protective standards and subsequently to estimate the risks of radon exposure in homes so as to develop guidelines for acceptable indoor concentrations [10]. The half-century of epidemiological research on the risks of radon in underground mines and in homes demonstrates the utility of observational studies for addressing societal questions on cancer risk and for guiding risk management approaches. Underground mines may have substantial levels of radon, which comes from the ore or from water in the mine. Radon is ubiquitous in homes, sometimes at concentrations as high as those measured in mines, but more often at far lower levels. The predominant source for indoor radon is soil gas containing radon produced by the natural decay of uranium.

Radon, the first occupational respiratory carcinogen to be identified, is an alpha-emitter that decays with a half-life of 3.5 days to a short-lived series of progeny [11]. Several of the short-lived progeny are also alpha-emitters and their alpha decays deliver the energy to target cells in the respiratory epithelium that is considered to cause radon-associated lung cancer [11]. Elegant laboratory research using single alpha particles demonstrates that one-hit of an alpha particle to a cell can lead to permanent change and also to "by-stander" effects on adjacent cells. The energy delivered to a cell from the alpha particles released by radon progeny is invariant with dose, reflecting the energy inherent to the decay process itself. These findings and dosimetric considerations have been interpreted as strongly supporting a no-threshold relationship between exposure and risk [11].

When the evidence on radon and cancer is considered within a causal assessment framework, the evidence was sufficient to identify radon as causally associated with lung cancer by the 1960s. By then, the substantial excess of lung cancer in radon-exposed Eastern European miners had been documented and the findings of several, more formal epidemiological studies of underground miners had been published, most notably the study of Colorado Plateau uranium miners in the United States [12]. Additionally, the dosimetry of radon progeny in the lung had been worked out and ionizing radiation generally had been established as a cause of cancer. Subsequently, the results of additional confirmatory epidemiological studies of underground miners were published [13]. In 1988, IARC reviewed the evidence, reaching the conclusion that radon and its decay products are a Group I carcinogen; as a member of the Working Group, I recall that this conclusion was reached quickly and without controversy, given the extensive human and biological evidence available [14].

The resulting epidemiological data were sufficiently robust to support analyses to develop epidemiologically-based risk models. As the epidemiological data strengthened, the models moved from being based in single studies to pooled data from multiple studies (for a description of these models, see the Biological Effects of Ionizing Radiation (BEIR) IV and BEIR VI reports of the US National Research Council [11,15]). These larger data sets facilitated the exploration of exposure-time-response relationships and the assessment of potential modifying factors. Evidence on radon and lung cancer became available from many different epidemiologic studies of underground miners including 11 studies that had quantitative exposure information suitable for pooled analysis to estimate the exposure-response relationship between exposure to radon progeny and lung cancer risk. These data were assembled in the early 1990s by Lubin et al. [16]; the pooled data set included more than 2,700 lung cancer deaths among 68,000 miners followed for nearly 1.2 million person-years of observation. A time-dependent risk model was developed by Lubin et al [16] and then extended by the BEIR VI Committee [11]. Conceptually, the BEIR VI Committee followed the approach used a decade earlier by the BEIR IV Committee, i.e., developing an empiric, time-dependent model for lung cancer risk from the miner data.

Most analyses were based on a linear excess relative risk (ERR) model:

RR = 1 + ßw or ERR = ßw,

where RR is relative risk, ß is a parameter measuring the unit increase in ERR per unit increase in w, and w is cumulative exposure to radon progeny. As in the BEIR IV analysis, ERR was linearly related to cumulative exposure to radon progeny without threshold. The ERR per unit exposure varied significantly with other factors; it decreased with attained age, time since exposure, and time after cessation of exposure but was not affected significantly by age at first exposure. Synergism with tobacco smoking was documented in analyses of data from the cohorts with smoking information available, but the interaction was submultiplicative. Over a wide range of total cumulative exposures to radon progeny, lung cancer risk increased as exposure rate declined, supporting the prior hypothesis of an inverse dose-rate effect [17]. The inverse dose-rate effect implies that the lower rates of exposure, typical of homes, could increase risk more than projected from estimates made at the generally higher exposures in mines. The extent of the information available at lower levels of exposure permitted analyses of risks in a range of exposures of greatest relevance to exposures associated with indoor radon. With the data restricted to the lower end of cumulative exposures, below 200 WLM, there was no evidence for departure from a linear model. The BEIR VI risk model was useful for estimating risks to miners and, by downward extrapolation, for estimating risks to the general population from indoor radon.

Because the first estimates of the lung cancer risk associated with indoor radon were based on the epidemiological studies of miners, the attendant uncertainties motivated epidemiological studies of radon and lung cancer in the general population. The initial studies were ecological and inherently limited such that multiple case-control studies were initiated subsequently. One ecological study, however, merits consideration because its findings were considered by some as countering the linear no-threshold models that had been applied to the miner data. The US ecological study carried out and extended periodically by Cohen [18,19] was widely cited across the 1980’s and 1990’s because of the finding of an inverse relationship between county-level radon average concentrations and lung cancer mortality. Critics of radon control programs referred to this study in characterizing the evidence on radon and lung cancer as too uncertain to warrant national programs [20]. However, the inherent flaws of the Cohen study were not acknowledged by either these critics or Cohen himself, although catalogued by the BEIR Committees and by Stidley and Samet in 1993 [21].

Cohen continued to publish ecological analyses on radon and lung cancer, using multivariate models with multiple factors in an attempt to control confounding [22]. An analysis by Puskin [23] provided strong indication of confounding by smoking. Puskin examined correlations of county mortality rates for other smoking-related cancers with county average radon levels and found negative correlations, as shown for lung cancer, a pattern indicating uncontrolled confounding by smoking. A review carried out by a scientific committee of the National Council on Radiation Protection and Measurements reached a similar conclusion [24]. The longstanding discussion of the Cohen analyses and their utilization in policy discussions reflects the willingness of some to use data that are flawed but consistent with a policy objective.

The initial wave of ecological studies was soon supplanted by case-control studies of lung cancer carried out in the general population to directly estimate the risk. The principal methodological problem in these studies was estimating domestic radon exposure during the relevant time span, possibly extending across the full lifespan. In some early studies, exposures were indirectly estimated based on surrogates such as type of residential construction or residence location [15]. In the more recent studies, exposures to indoor radon were estimated by making longer-term measurements of radon concentration in the current and previous residences of the cases and controls [11]. Several of the studies have been restricted to nonsmokers or long-term former smokers in order to estimate the lung cancer risk in this group as precisely as possible. A substantial number of these case-control studies were carried out in North America and Europe and the results reported from the 1980’s forward.

As the initial findings were reported and new studies were initiated, potential limitations of the individual studies were reported. A need for pooling of study results was predicted based on sample size calculations published by Lubin *et al.* in 1990 [25]. The sample size calculations considered the consequences of measurement error and residential mobility, which together tend to reduce the variability of population exposure. The calculations indicated that the total sample size needed for hypotheses of interest approached the total number of cases from the completed and the then ongoing studies, approximately 15,000. An update of these calculations [26] confirmed the need for large sample sizes and this recognition led to a prospective plan for pooling of the various studies to achieve the maximum statistical power.

**Figure 2:**
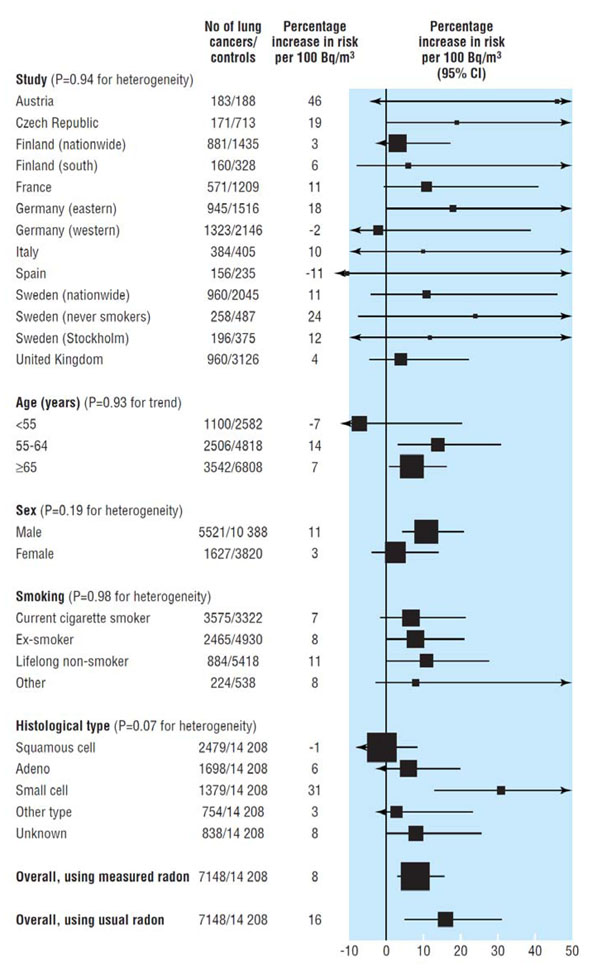
Increase in lung cancer risk per 100 Bq m^-3^ increase in measured radon concentration in 13 European case-control studies Source: Darby et al. 2005 [28]

In 2005, the results of pooled analyses of data from North America [27] and from Europe [28] were reported. The North American analysis included data from seven studies with 3,662 cases and 4,966 controls. The risk of lung cancer was estimated to increase by 11% (95% CI 0-28%) per 100 Bq m^-3^ increment in the concentration at which exposure occurs in a home. The estimate from the pooling of 13 European studies involving 7,148 cases and 14,208 controls was similar: an increment of 8% (95% CI 3-16%) per 100 Bq m^-3^ increment in home concentration was estimated. The estimates from the two pooled analyses are comparable and are close to estimates projected models based in the data from underground miners. There was no indication that the risk of radon varied across a number of potential modifiers, such as age and smoking (Figure 2). The global pooling will be completed within the year.

Thus, the evidence on radon and lung cancer risk is substantial and coherent and indoor radon is widely considered to be carcinogenic. Nonetheless, some remain skeptical, perhaps primarily because of the adoption of a no-threshold relationship between lung cancer risk and radon exposure. Critics of this model and of radon control initiatives have turned to the results of one recent study as providing countering evidence. In a case-control study in Massachusetts, Thompson and colleagues [29] estimated exposure to radon for 200 cases and 397 controls; overall, they did not find an association between estimated radon exposure and lung cancer risk and, in fact, the risk dipped at the higher levels. In spite of the already accumulated epidemiological evidence, including the two pooled analyses and the strong biophysical basis for radon-induced carcinogenesis at any level of exposure, the results of this study were used to question the linear, no-threshold relationship of radon with lung cancer risk [30,31]. In fact, Scott et al. stated “Based on the results presented, we can therefore state that for people residing in homes with a radon concentration in the range 25–250 Bq m^−3^ (0.676 − 6.76 pCi L^−1^), *remediation of their homes to eliminate radon may lead to a substantial increase in their lung cancer risk*!”[30].

The example of radon and lung cancer provides a number of "lessons learned" concerning the use of epidemiological evidence to control cancer. First, the identification of unacceptable levels of lung cancer risk was readily identified in single cohorts of highly exposed underground miners. Second, as exposures came under control, there was a need to characterize the exposure-response relationship so as to set workplace standards with an acceptable level of risk. The individual studies did not provide a robust data base for this purpose, but the pooled data bases did. Third, as for other exposures, extrapolations of the exposure-response relationship observed in underground miners to the general population were subject to substantial uncertainty and that uncertainty entered into policy discussions. Fourth, the prospective plan for pooling of the indoor radon studies led to the most informative data base possible from the diverse under-powered studies that were implemented. Fifth, integrative syntheses of epidemiological evidence with other research findings, as carried out by the BEIR Committees, are critical for robust interpretation of epidemiological evidence. Sixth, even very strong evidence and scientific consensus may not quiet all critics, particularly those motivated by the policy implications of the scientific evidence. In the example of radon, critics continued to claim that uncertainties abound, even as the evidence became firmer [32].

## The continuing need for epidemiological research

Turning to radiation generally, a century after we learned that radiation can cause cancer and with a half-century of accumulated observational evidence on the risks of radiation, why is more research needed? Two main reasons can be cited: 1) the need for an ever stronger base of evidence on the risks of radiation to protect the public and workers from unnecessary risk of cancer; and 2) the exposure of the population to new forms of radiation for which information on risks is still uncertain. Additionally, the emergence of genomics offers the possibility of identifying genetic determinants of risk, leading to the prospect of identifying those at high risk for cancer caused by therapeutic irradiation. Additionally, debate about the nature of the cancer risk associated with radiation has become almost ideological on some matters, such as the nature of the dose-response relationship, and further evidence may help to bring this distracting debate to a close.

The "low-dose" controversy persists and will likely remain, absent a new level of mechanistic understanding that identifies the biophysical process underlying radiation carcinogenesis with even greater certainty. Even for radon progeny for which a high level of certainty on the mechanism of DNA damage by alpha particles has been reached, there is still debate on risks at lower levels. A recent point-counterpoint in *Radiology* is illustrative. Little and colleagues [33], under the title--"Risks Associated with Low Doses and Low Dose Rate of Ionizing Radiation: Why Linearity May be (Almost) the Best That We Can Do"--conclude that the evidence on biological mechanisms weighs against any possible hormesis and the existence of a threshold. A similar conclusion was reached in the 2003 in an evidence synthesis carried out by leading researchers on the topic [34]. Tubiana and colleagues [35] in their counterpoint entitled----"The Linear No-Threshold Relationship Is inconsistent with Radiation Biologic and Experimental Data"--find indication of hormesis and evidence against a no-threshold relationship. They propose that repair mechanisms are in-place and that adaptive responses are beneficial, i.e., hormetic. They view the epidemiological evidence as failing to demonstrate carcinogenesis at lower doses of low linear energy transfer radiation. Their arguments move to the policy and specific radiation protection implications of a risk relationship that is linear without threshold.

Further epidemiological research is unlikely to bring this now-lengthy discussion to closure. At lower doses, even large data sets are not informative as to alternative forms of the exposure-response relationship. Additionally, as the range of evidence extends to lower doses, critics will likely continue to claim uncertainty, but at ever lower doses. The combination of more robust epidemiological evidence and deepening mechanistic understanding may eventually silence critics, as has apparently occurred with radon.

The more recent debate concerning potential risks of high frequency electromagnetic radiation from cell phones illustrates another need for further research: the emergence of a nearly ubiquitous exposure with uncertain consequences. Cell phone ownership has surged over the last 20 years, with worldwide penetration. As the technology and making calls have become cheaper, usage has surged. Children now have cell phones from an early age and ever-increasing amounts of time are spent using them. Public concern about the possibility of carcinogenesis by electromagnetic radiation generally, like that emitted by cell phones is not new [36-39]. From the mid-1970s, studies had been carried out on low frequency electromagnetic radiation generated by power lines and most notably childhood leukemias, but on other malignancies as well [36,39]. For cell phones, the concern has been specifically with brain cancer, since the brain is exposed to electromagnetic radiation during cell phone use. Other sites are of concern as well, including the acoustic nerve and salivary glands, but there are fewer studies of those sites because of the low incidence rates for these malignancies.

Even in the 1990s, although exposures of users were of relatively brief duration, epidemiological studies were initiated. The case-control design was used to compare cell phone use of people with brain cancer with use by controls. Inskip and colleagues, for example, at the U.S. National Cancer Institute carried out a multi-site case-control study that was reported in the *New England Journal of Medicine*[40]. The International Agency for Research on Cancer (IARC) organized a multisite study, the INTERPHONE study, involving 13 countries [41]. The overall findings were published in June 2010 [42], although some of the component sites had reported their findings (see selected articles by INTERPHONE groups in Sweden [43], Denmark [44], Germany [45], the U.K. [46], and Norway [47]). The findings of the component sites provided mixed evidence; publication of the long-awaited multi-site findings also left the question open as to whether cell phones cause brain cancer [42,48].

Further research is also needed because of the potential for "surprises". For example, the Chernobyl disaster was shortly followed by an epidemic of thyroid cancer among children, an epidemic that was not anticipated based on then extent epidemiological data and knowledge of the radionuclides to which the children were exposed [49]. Changing patterns of medical exposure are also of concern, as diagnostic imaging is more widely applied and at young ages. Notably, medical irradiation now comprises the largest proportion of the radiation received by the US population, surpassing radon [8]. For some groups, such as children with cystic fibrosis, medical monitoring with CAT scans may lead to sustained exposure from an early age [50].

Finally, there is the major scientific opportunity for identifying genes that increase risk for radiation carcinogenesis. Investigation of gene X environment interaction is a burgeoning area of research. Compared with some other environmental agents to which the public is broadly exposed, radiation exposure is measured more feasibly and with greater accuracy. Studies of therapeutic exposures have the strength of a precisely administered and recorded dose. The BEIR VII report, the last review by the US National Research Council, pointed to genetics as an area for further research [51].

Looking ahead, consideration needs to be given to the best models for carrying out radiation epidemiology research. The research is inherently multidisciplinary, drawing on the expertise not only of epidemiologists, but biostatisticians and risk assessors, health physicists, and radiation biologists. Studies on the genetic basis of susceptibility need additional expertise in genetic epidemiology and genomics. Epidemiological research on radiation risks is centralized in a small number of centers worldwide, including the Radiation Epidemiology Branch of the US National Cancer Institute, the Radiation Effect Research Foundation, and IARC, and several universities. The field needs to be maintained and strengthened and transitioned beyond an emphasis on quantification of risk to integration of genetics in the search for genes affecting cancer risk associated with radiation. Epigenetic approaches may also prove useful. Surveillance approaches based in administrative systems are also needed to track the consequences of changing patterns of exposure to medical radiation.

## Concluding remarks

While the identification of cigarette smoking as a cause of lung cancer is touted as one of the key, early successes of epidemiological research, cohort studies were documenting the risk of cancer associated with radiation during the same decades--the 1950s and 1060s. The need to move quickly from hazard identification to dose-response assessment to support radiation standards development motivated the application of quantitative models to the epidemiological data. The data from the LifeSpan Study of Atomic Bomb Survivors provided an early opportunity for dose-response modeling and the exploration of dose-response relationships over time.

The example of radiation points to one potential failing of the IARC Monograph series for control of environmental carcinogens: the purpose is to assess carcinogenicity and classify the strength of evidence for causation, and not to quantify risk associated with a carcinogen. However, regulation of many carcinogens is increasingly based in quantitative risk models. This restriction of the IARC approach is well recognized, as are the consequences. A move to developing risk models would necessitate a different approach by IARC, and possibly the addition of a group within the Agency that could carry out the modeling. If IARC were to move in this direction, building on the legacy of Lorenzo Tomatis, radiation would be the right starting point.

## Competing interests

The author declare no competing financial or non-financial interests.

**Table 1:** Radiation agents reviewed in the International Agency for Research on Cancer's (IARC) monograph series

Agent	Group	IARC Monograph Volume No.	Year
Ultraviolet radiation	1	40, 55	1986, 1992
Radon-222 and its decay products	1	43, 78	1988, 2001
Ultraviolet radiation A (NB: Overall evaluation upgraded from 2B to 2A with supporting evidence from other relevant data)	2A	55	1992
Ultraviolet radiation B (NB: Overall evaluation upgraded from 2B to 2A with supporting evidence from other relevant data)	2A	55	1992
Ultraviolet radiation C (NB: Overall evaluation upgraded from 2B to 2A with supporting evidence from other relevant data)	2A	55	1992
Solar radiation	1	55	1992
X- and Gamma (γ)-Radiation	1	75	2000
Radium-224 and its decay products	1	78	2001
Radium-226 and its decay products	1	78	2001
Radium-228 and its decay products	1	78	2001
Radioiodines, short-lived isotopes, including iodine-131, from atomic reactor accidents and nuclear weapons detonation (exposure during childhood)	1	78	2001
Radionuclides, α-particle-emitting, internally deposited (NB: Specific radionuclides for which there is sufficient evidence for carcinogenicity to humans are also listed individually as Group 1 agents)	1	78	2001
Radionuclides, β-particle-emitting, internally deposited (NB: Specific radionuclides for which there is sufficient evidence for carcinogenicity to humans are also listed individually as Group 1 agents)	1	78	2001
Magnetic fields (extremely low-frequency)	2B	80	2002
Magnetic fields (static)	3	80	2002

Classification of carcinogenic hazards to humans:

Group 1: Carcinogenic to humans

Group 2A: Probably carcinogenic to humans

Group 2B: Possibly carcinogenic to humans

Group 3: Not classifiable as to carcinogenicity to humans

Group 4: Probably not carcinogenic to humans

## References

[B1] March HC Leukemia in radiologists Radiology 1944 43 275 278

[B2] Hacker BC The dragon's tail : radiation safety in the Manhattan Project, 1942-1946 1987 Berkeley: University of California Press

[B3] Schull WJ Effects of atomic radiation. A half-century of studies from Hiroshima and Nagasaki 1995 New York: Wiley-Liss

[B4] Radiation Effects Research Foundation (RERF) http://www.rerf.or.jp/index_e.html

[B5] National Research Council Science and decisions: advancing risk assessment 2009 Washington, D.C.: National Academies Press 25009905

[B6] National Research Council Committee on the Institutional Means for Assessment of Risks to Public Health Risk Assessment in the Federal Government: Managing the Process 1983 Washington, D.C.: National Academy Press

[B7] Lowrance WW Of Acceptable Risk: Science and the Determination of Safety 1976 Los Altos, CA: William Kaufmann, Inc.

[B8] National Council on Radiation Protection and Measurements Ionizing radiation exposure of the population of the United States : recommendations of the National Council on Radiation Protection and Measurements 2009 Bethesda, Md.: National Council on Radiation Protection and Measurements

[B9] Cook R Calabrese EJ The importance of hormesis to public health Environ Health Perspect 2006 114 11 1631 1635 17107845 10.1289/ehp.8606 PMC1665397

[B10] Samet JM McQueen CA Radon Comprehensive Toxicology 2010 2 New York: Elsevier

[B11] National Research Council (U.S.) Committee on Health Risks of Exposure to Radon Health effects of exposure to radon: BEIR VI 1999 Washington, D.C.: National Academy Press

[B12] Wagoner JK Archer VE Lundin FE Jr. Holaday DA Lloyd JW Radiation as the cause of lung cancer among uranium miners New England Journal of Medicine 1965 273 181 188 10.1056/NEJM196507222730402 14306333

[B13] Samet JM National Council on Radiation Protection and Measurements Epidemiological studies of lung cancer in underground miners Radon Proceedings of the Twenty-Fourth Annual Meeting of the National Council on Radiation Protection and Measurements 1988 Bethesda, Maryland: NCRP 30 50

[B14] International Agency for Research on Cancer IARC Monographs on the Evaluation of Carcinogenic Risks to Humans. Man-made Mineral Fibers and Radon. Vol 43 1988 Lyon, France: World Health Organization, IARC

[B15] National Research Council Committee on the Biological Effects of Ionizing Radiation Health Risks of Radon and Other Internally Deposited Alpha-Emitters: BEIR IV 1988 Washington, D.C.: National Academy Press 25032289

[B16] Lubin JH Boice JD Jr. Edling C Hornung RW Howe G Kunz E Kusiak RA Morrison HI Radford EP Samet JM Radon and Lung Cancer Risk: A Joint Analysis of 11 Underground Miners Studies 1994 Bethesda, Maryland: U.S. Department of Health and Human Services, Public Health Service, National Institutes of Health

[B17] Lubin JH Boice JD Jr. Edling C Hormung RW Howe G Kunz E Kusiak RA Morrison HI Radford EP Samet JM Radon-exposed underground miners and inverse dose-rate (protraction enhancement) effects Health Physics 1995 69 4 494 500 10.1097/00004032-199510000-00007 7558839

[B18] Cohen BL Test of the linear-no threshold theory of radiation carcinogenesis for inhaled radon decay products Health Physics 1995 68 2 157 174 10.1097/00004032-199502000-00002 7814250

[B19] Cohen BL Colditz GA Tests of the linear-no threshold theory for lung cancer induced by exposure to radon Environmental Research 1994 64 1 65 89 10.1006/enrs.1994.1007 8287843

[B20] Cole LA Elements of risk: the politics of radon 1993 Washington, D.C.: AAAS Press

[B21] Stidley CA Samet JM A review of ecologic studies of lung cancer and indoor radon Health Physics 1993 93 3 234 251 10.1097/00004032-199309000-00001 8244693

[B22] Cohen BL Cancer risk from low-level radiation AJR Am J Roentgenol 2002 179 5 1137 1143 12388487 10.2214/ajr.179.5.1791137

[B23] Puskin JS Smoking as a confounder in ecologic correlations of cancer mortality rates with average county radon levels Health Phys 2003 84 4 526 532 10.1097/00004032-200304000-00012 12705451

[B24] Heath CW Jr. Bond PD Hoel DG Meinhold CB Residential radon exposure and lung cancer risk: commentary on Cohen's county-based study Health Phys 2004 87 6 647 655 10.1097/01.HP.0000138588.59022.40 15545771

[B25] Lubin JH Samet JM Weinberg C Design issues in epidemiologic studies of indoor exposure to radon and risk of lung cancer Health Physics 1990 59 807 817 10.1097/00004032-199012000-00004 2228608

[B26] Lubin JH Boice JD Jr. Samet JM Errors in exposure assessment, statistical power and the interpretation of residential radon studies Radiat Res 1995 144 3 329 341 10.2307/3578953 7494877

[B27] Krewski D Lubin JH Zielinski JM Alavanja M Catalan VS Field RW Klotz JB Letourneau EG Lynch CF Lyon JI Residential radon and risk of lung cancer: a combined analysis of 7 North American case-control studies Epidemiology 2005 16 2 137 145 10.1097/01.ede.0000152522.80261.e3 15703527

[B28] Darby S Hill D Auvinen A Barros-Dios JM Baysson H Bochicchio F Deo H Falk R Forastiere F Hakama M Radon in homes and risk of lung cancer: collaborative analysis of individual data from 13 European case-control studies British Medical Journal 2005 330 7485 223 10.1136/bmj.38308.477650.63 15613366 PMC546066

[B29] Thompson RE Nelson DF Popkin JH Popkin Z Case-control study of lung cancer risk from residential radon exposure in Worcester county, Massachusetts Health Phys 2008 94 3 228 241 10.1097/01.HP.0000288561.53790.5f 18301096

[B30] Scott BR Belinsky SA Leng S Lin Y Wilder JA Damiani LA Radiation-Stimulated Epigenetic Reprogramming of Adaptive-Response Genes in the Lung: An Evolutionary Gift for Mounting Adaptive Protection Against Lung Cancer Dose Response 2009 7 2 104 131 19543479 10.2203/dose-response.08-016.Scott PMC2695570

[B31] Vaiserman AM Radiation hormesis: historical perspective and implications for low-dose cancer risk assessment Dose Response 2010 8 2 172 191 10.2203/dose-response.09-037.Vaiserman 20585444 PMC2889502

[B32] Kabat GC Hyping health risks: environmental hazards in daily life and the science of epidemiology 2008 New York: Columbia University Press

[B33] Little MP Wakeford R Tawn EJ Bouffler SD Berrington de Gonzalez A Risks associated with low doses and low dose rates of ionizing radiation: why linearity may be (almost) the best we can do Radiology 2009 251 1 6 12 10.1148/radiol.2511081686 19332841 PMC2663578

[B34] Brenner DJ Doll R Goodhead DT Hall EJ Land CE Little JB Lubin JH Preston DL Preston RJ Puskin JS Cancer risks attributable to low doses of ionizing radiation: assessing what we really know Proc Natl Acad Sci USA 2003 100 24 13761 13766 10.1073/pnas.2235592100 14610281 PMC283495

[B35] Tubiana M Feinendegen LE Yang C Kaminski JM The linear no-threshold relationship is inconsistent with radiation biologic and experimental data Radiology 2009 251 1 13 22 10.1148/radiol.2511080671 19332842 PMC2663584

[B36] Wertheimer N Leeper ED Electrical wiring configurations and childhood cancer American Journal of Epidemiology 1979 109 273 284 453167 10.1093/oxfordjournals.aje.a112681

[B37] Brodeur P The great power-line cover-up : how the utilities and the government are trying to hide the cancer hazard posed by electromagnetic fields 1993 1 Boston: Little, Brown and Co.

[B38] Brodeur P Currents of death : power lines, computer terminals, and the attempt to cover up their threat to your health 1989 New York: Simon and Schuster

[B39] National Research Council Possible health effects of exposure to residential electric and magnetic fields 1997 Washington, DC: National Academy Press 25121270

[B40] Inskip PD Tarone RE Hatch EE Wilcosky TC Shapiro WR Selker RG Fine HA Black PM Loeffler JS Linet MS Cellular-telephone use and brain tumors N Engl J Med 2001 344 2 79 86 10.1056/NEJM200101113440201 11150357

[B41] Cardis E Richardson L Deltour I Armstrong B Feychting M Johansen C Kilkenny M McKinney P Modan B Sadetzki S The INTERPHONE study: design, epidemiological methods, and description of the study population Eur J Epidemiol 2007 22 9 647 664 10.1007/s10654-007-9152-z 17636416

[B42] Brain tumour risk in relation to mobile telephone use: results of the INTERPHONE international case-control study Int J Epidemiol 2010 39 3 675 694 10.1093/ije/dyq079 20483835

[B43] Lonn S Ahlbom A Hall P Feychting M Long-term mobile phone use and brain tumor risk Am J Epidemiol 2005 161 6 526 535 10.1093/aje/kwi091 15746469

[B44] Christensen HC Schuz J Kosteljanetz M Poulsen HS Boice JD Jr. McLaughlin JK Johansen C Cellular telephones and risk for brain tumors: a population-based, incident case-control study Neurology 2005 64 7 1189 1195 15824345 10.1212/01.WNL.0000156351.72313.D3

[B45] Schuz J Bohler E Berg G Schlehofer B Hettinger I Schlaefer K Wahrendorf J Kunna-Grass K Blettner M Cellular phones, cordless phones, and the risks of glioma and meningioma (Interphone Study Group, Germany) Am J Epidemiol 2006 163 6 512 520 10.1093/aje/kwj068 16443797

[B46] Hepworth SJ Schoemaker MJ Muir KR Swerdlow AJ van Tongeren MJ McKinney PA Mobile phone use and risk of glioma in adults: case-control study BMJ 2006 332 7546 883 887 10.1136/bmj.38720.687975.55 16428250 PMC1440611

[B47] Klaeboe L Blaasaas KG Tynes T Use of mobile phones in Norway and risk of intracranial tumours Eur J Cancer Prev 2007 16 2 158 164 10.1097/01.cej.0000203616.77183.4c 17297392

[B48] Saracci R Samet J Commentary: Call me on my mobile phone...or better not?--a look at the INTERPHONE study results Int J Epidemiol 2010 39 3 695 698 10.1093/ije/dyq082 20483832 PMC2878457

[B49] Moysich KB Menezes RJ Michalek AM Chernobyl-related ionising radiation exposure and cancer risk: an epidemiological review Lancet Oncol 2002 3 5 269 279 10.1016/S1470-2045(02)00727-1 12067803

[B50] de Gonzalez AB Kim KP Samet JM Radiation-induced Cancer Risk from Annual Computed Tomography for Patients with Cystic Fibrosis Am J Respir Crit Care Med 2007 176 970 973 10.1164/rccm.200704-591OC 17717201

[B51] National Research Council Health Risks from Exposure to Low Levels of Ionizing Radiation: BEIR VII Phase 2 2006 Washington, DC: National Academy of Sciences 25077203

